# Utilizing Gamification, Artificial Intelligence, and mHealth for the Professional Development of Maternal Care Providers: Exploratory Pilot Cross-Sectional Study Assessing Providers' Satisfaction in Primary Health Care Centers in Lebanon

**DOI:** 10.2196/53735

**Published:** 2025-09-10

**Authors:** Mohamad Alameddine, Nadine Sabra, Nour El Arnaout, Asmaa El Dakdouki, Mahmoud El Jaouni, Randa Hamadeh, Abed Shanaa, Shadi Saleh

**Affiliations:** 1 College of Health Sciences, University of Sharjah, Sharjah, United Arab Emirates; 2 Global Health Institute, American University of Beirut, PO Box 11-0236, Riad El Solh, Beirut, 1107 2020, Lebanon, 961 3047578; 3 Faculty of Health Sciences, American University of Beirut, Beirut, Lebanon; 4 Ministry of Public Health, Beirut, Lebanon; 5 The United Nations Relief and Works Agency for Palestine Refugees in the Near East, Beirut, Lebanon; 6 Department of Health Management and Policy, Faculty of Health Sciences, American University of Beirut, Beirut, Lebanon

**Keywords:** maternal health, gamification, artificial intelligence, professional development, mHealth, health provider, medical knowledge

## Abstract

**Background:**

High maternal morbidity and mortality rates globally, especially in low-income and lower-middle–income countries, highlight the critical role of skilled health care providers (HCPs) in preventing pregnancy-related complications among disadvantaged populations. Lebanon, hosting over 1.5 million refugees, is no exception. HCPs face significant challenges, including resource constraints and limited professional development opportunities, underscoring the need for continuous learning and innovative educational interventions. Artificial intelligence (AI) and gamification show promise in enhancing clinical performance and evidence-based practice.

**Objective:**

Considering the limited evidence on the effectiveness of integrating gamification and AI in a mobile app for professional development of HCPs providing maternal health services, this pilot study aims to assess the satisfaction and acceptability of HCPs with a novel mLearning tool, titled the “GAIN MHI” app (gamification, artificial intelligence, and mHealth network for maternal health improvement), at selected primary health care centers in Lebanon.

**Methods:**

This is a cross-sectional study that presents data collected from 12 participating HCPs, primarily obstetricians and midwives who have been using the GAIN MHI mobile app for professional development and learning. The survey used included Likert scale questions to assess HCPs’ satisfaction, engagement, and evaluation of the gamification and AI components of the app. Open-ended questions gathered qualitative feedback on app preferences and potential improvements. Statistical analysis was performed to derive insights from the quantitative data collected. Subsequently, a descriptive analysis was performed, presenting the frequencies and percentages of various participant characteristics, as well as responses to the survey across all sections.

**Results:**

A total of 85% (n=10) of the HCPs, including midwives and doctors, were satisfied with the GAIN MHI mobile app, the user interface, and various content features. Engagement levels were robust (64.6%, SD 6.2%), notably impacting clinical routines and theoretical knowledge. The gamification and AI components garnered strong positive feedback, enhancing learning enjoyment (11/12, 92%). From a qualitative perspective, users expressed appreciation for the app’s diverse content, user-friendliness, and motivation for continuous learning. Suggestions for expanding the content included a wide range of health topics, highlighting the app’s potential applicability in various health care fields.

**Conclusions:**

HCPs, especially those practicing in underserved areas, face challenges in accessing professional development opportunities, highlighting the need for innovative pedagogical approaches using mobile technologies. This pilot study underlines the potential of using AI-based digital solutions for professional development with the aim of improving the quality of health services—in this case, maternal health services—through continuous learning and updates on the most recent evidence-based clinical guidelines. Future research should investigate the feasibility of applying similar solutions on a larger scale to reach a wider range of HCPs and cover other health topics. The applicability of such solutions in different contexts and low-resource settings should also be explored.

## Introduction

### Overview of Maternal Health Status on Global and National Levels

Globally, around 800 women die on a daily basis from preventable causes related to pregnancy and childbirth, with 95% of the deaths occurring in low and lower-middle-income countries [1]. In the context of Lebanon, where women of childbearing age constitute the majority of the refugee population, increased maternal mortality rates and pregnancy-related complications are documented, particularly among socioeconomically disadvantaged populations [2-5]. In terms of maternal health services utilization, the administration of care by proficient health care providers (HCPs) prior to, during, and postpartum holds the potential to mitigate mortality rates among women and neonates [1].

### Health Care Providers: Roles, Challenges, and Pathways to Improvement in Maternal Health

HCPs play a vital role within the health care system, addressing a wide range of matters such as maternal health, family planning, neonatal care, child nutrition, and vaccination [6]. It is essential to acknowledge that HCPs encounter various challenges in fulfilling their roles [7,8]. Resource constraints, including limited continuing education programs and career development, a shortage in health care professionals, and disparities in geographic distribution of providers’ care, can hinder their efforts, particularly in Lebanon [9-11]. A promising approach to enhance the clinical performance and the evidence-based practice of HCPs is advocating for continuous learning and professional development [12,13]. The foregoing could be attained by developing innovative approaches and educational interventions that ensure their enduring involvement [13,14].

### Existing Innovative Approaches and Educational Interventions for Maternal Health Targeting Health Care Providers

Clinical competence among HCPs plays a pivotal role in ensuring the quality of care [15]. Lifelong learning serves as an indispensable component of practice-based learning and improvement for HCPs, as it directly contributes to maintaining and enhancing their clinical competence [16]. The dynamic nature of health care requires providers to stay informed about the latest medical advancements, evidence-based practices, and technological innovations [17,18]. Considering the expenses and practical difficulties associated with delivering in-person continuing education training programs for HCPs, the use of mobile phones and comparable portable electronic devices emerges as a promising approach [19,20], commonly referred to as mobile learning or mLearning [21]. This approach effectively extends the reach of continuing education programs to distant health care workers, providing them with current and relevant information [19]. Only a limited number of research endeavors have specifically addressed the use of mobile phones to enhance the knowledge and competencies of HCPs in maternal health in low- and middle-income countries [19-22]. One example of the mLearning interventions is the Safe Delivery App (SDA), which was developed to enhance the proficiency and skills of HCPs through visually animated instruction videos and guiding steps for effectively addressing fundamental obstetric and neonatal emergencies, particularly in low-resource settings. The SDA app was successfully implemented in the Democratic Republic of the Congo [21] and was subsequently implemented [21,23] in some countries of the Middle East and North Africa region [24]. In order to maximize the advantages of the SDA app, an upgraded version was developed in India, incorporating gamification elements to enhance the motivation of the users while transforming the learning process into an interactive game [25].

### Gamification and Artificial Intelligence for Health Care Providers in the Field of Maternal Health

Over the past few years, there has been a notable increase in global recognition regarding the application of gamification across various domains [26]. Gamification, defined as “the practice of introducing ‘game-like’ dynamics into routine activities to engage users” [22], holds the promise of enabling HCPs to engage in dynamic learning, tackle clinical challenges, and garner experience within a secure and controlled environment [22,23]. Gamification is characterized by the incorporation of game design elements into non-game-related processes, with inclusion of badges, points, and scoring systems [26-28]. Given the relatively new nature of the field, research is essential to establish evidence-based practices, guidelines, and standards for gamification and its integration in mobile health (mhealth) apps [28]. Exploring gamification methods through research can assist in identifying the best game design elements for diverse contexts and populations and integrating innovative approaches into existing systems [28]. Besides gamification, the use of artificial intelligence (AI) in mHealth apps is being investigated, with the goal of delivering health care that is more personalized [29,30] and improving overall health care provision [31,32]. AI refers to the capacity of systems to analyze data and uses computers and machines to amplify human decision-making, problem-solving skills, and the innovation driven by technology [33-35]. Within the realm of clinical practice, AI aids the HCPs in making diagnoses and treatment choices [36,37], by providing them with specialized knowledge and skills [38]. As for maternal health, studies showed that AI may reduce the time needed to train HCPs in evaluating preterm deliveries [39], and help in predicting different maternal complications such as gestational diabetes [40,41] and postpartum depression [42,43]. There is clear evidence of AI’s impact extending to nearly every facet of the health care industry [44], yet the integration of AI with gamification in mHealth apps is still a novel and evolving concept.

### Study Aim

Considering the limited evidence on integrating gamification and AI in a mobile app targeting the HCPs in maternal health, this pilot study aims to assess the satisfaction and acceptability of HCPs with a novel mLearning tool, titled the “GAIN MHI” app (gamification, artificial intelligence, and mHealth network for maternal health improvement), at selected primary health care centers (PHCs) in Lebanon.

## Methods

### The GAIN MHI App

**Figure 1.**
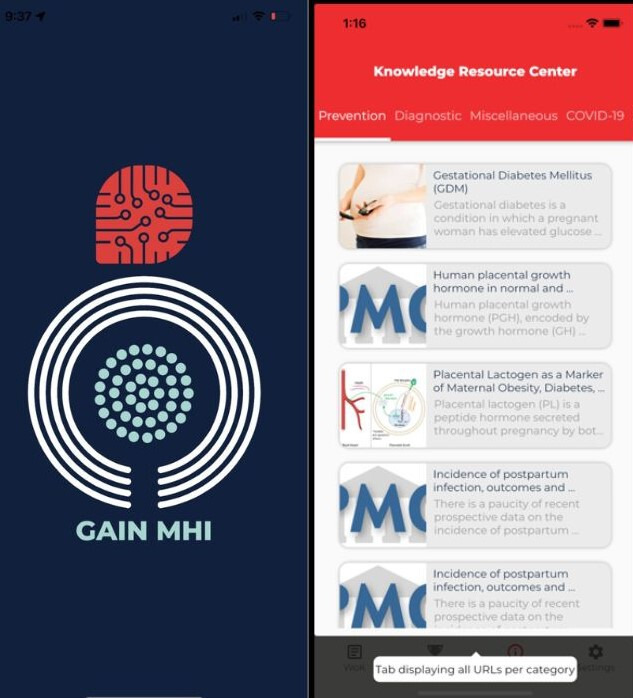
The GAIN MHI mobile app user interface. GAIN MHI: gamification, artificial intelligence, and mHealth network for maternal health improvement.

The GAIN MHI app (Figure 1) is a mobile app developed by the Global Health Institute at the American University of Beirut as part of the GAIN MHI project. Through this mobile app, the project aims to improve the quality of care provided to pregnant women by their specialized HCPs (mainly obstetrics and gynecology [OBGYN] specialists and midwives). The GAIN MHI mobile app was characterized by 2 components: the gamification of learning and the AI components, representing a novel intervention for the professional development of the HCPs. Gamification of learning is defined within the context of this project as the app of game principles and design elements for the purpose of professional development. The gamification component in the GAIN MHI mobile app included “trivia-like” concept and monthly recognition as most valuable player and most improved player (MIP), coupled with monetary incentives equivalent to US $150 each. The questions in the app fell under 5 [5] categories related to maternal health: Prevention, Diagnostic, Management, Miscellaneous, and COVID-19 related questions. The category selection process relies on a randomized spin of a wheel, and each user is allocated a maximum of 30 attempts per month. Consequently, users are required to respond to 30 questions within the given monthly timeframe.

The project, through the GAIN MHI mobile app, used AI algorithms to identify areas with knowledge gaps based on the incorrect answers of health providers. The question generation formula encompassed both personalization and diversity. This allowed the tailoring of knowledge shared in subsequent questions to make the latter individualized to each provider based on their performance on the multiple-choice questions (MCQs). To this end, a repository of questions was built. Topic modeling was used on the repository. This allowed the identification of the topic of wrongly answered questions. Explanations were provided after answering each MCQ. Further, the application was coupled with automated WhatsApp messages sent to the users regarding their score, right/wrong answers, remaining spins for the month, monthly performance, and any gained rewards. The content of the app was available in both English and Arabic languages.

### Project Implementation, Population, and Setting

The GAIN MHI app database was created by a medical consultant specializing in OBGYN. The database incorporated questions focused on antenatal and postnatal care practice guidelines targeting OBGYN physicians, questions focused on antenatal and postnatal care practice guidelines targeting midwives, corresponding options, correct answers, explanations, and resources for references. To cater to the Arabic-speaking HCPs, an Arabic version of the app was generated. Subsequently, a validation process was performed in which questions dedicated to OBGYNs and midwives were validated by specialists from the same field. The aim was to ensure that the database content was tailored to all intended users. After the validation, the intervention’s components were eventually adjusted based on the participants’ inputs and suggestions. Additionally, a testing phase was conducted to guarantee the satisfaction of users with the look, feel, and usability of the mobile app. Based on that, some features of the app were upgraded as per the shared feedback.

This study took place over a period of 21 months (from September 2021 to May 2023), in 6 PHCs located in different governorates of Lebanon, including South, Beirut, and Mount Lebanon. Among these PHCs, 3 were affiliated with the United Nations Relief and Works Agency and the others with the Ministry of Public Health. A total of 15 HCPs (OBGYN specialists and midwives) in maternal health were enrolled in the intervention, of which 9 were midwives and 6 were OBGYN specialists. At the termination of the study, 12 participants (8 midwives, 67% and 4 OBGYN specialists, 33%) maintained their participation, whereas 3 participants dropped out. Among those who continued, 67% (n=8) were female and 33% (n=4) were male. Regarding language preferences, 10 participants used the app in English while 2 participants used it in Arabic (Table 1).

**Table 1.** General characteristics of participants (N=12): frequency and percentage distribution.

Variables	Values, n (%)
PHC^a^ affiliation	
MOPH^b^	6 (50)
UNRWA^c^	6 (50)
Title	
Midwife	8 (67)
Doctor	4 (33)
Sex	
Female	8 (67)
Male	4 (33)
Language of app use	
English	10 (83)
Arabic	2 (17)

a PHC: primary health care center.

b MOPH: Ministry of Public Health.

c UNRWA: United Nations Relief and Works Agency.

### Data Collection

In this study, both quantitative and qualitative research methods were used to comprehensively assess the GAIN MHI app, providing numerical insights and in-depth feedback concerning this app. A cross-sectional survey was designed considering the features of the GAIN MHI app. It included Likert scale and open-ended questions to gather both quantitative rates and qualitative perceptions of the app users, respectively. The 5-point Likert scale was used to assess the level of satisfaction of HCPs with the characteristics of the app, their level of engagement, and their evaluation of the Gamification and the AI components of the app. The scale consists of the following points: highly dissatisfied [1], dissatisfied [2], neither satisfied nor dissatisfied [3], satisfied [4], and highly satisfied [5]. Complementary to this, the open-ended questions explored feedback from the HCPs on the favorite aspect of the mobile app, preferred types of reward, maternal health topics that should be added to the app, health topics other than obstetrics/gynecology where this app could be applied, as well as suggestions for future improvement. Together, the quantitative and qualitative methods reveal the satisfaction with the app and identify potential areas for enhancement. The survey was filled out in person with the 12 participants after signing a written consent form.

To ensure the reliability of the questionnaire, Cronbach α (*α*=0.82) was calculated, indicating high internal consistency. Additionally, reliability was assessed for each subscale, with results of 0.74, 0.64, and 0.8 reflecting acceptable, moderate, and high reliability among the 3 sections, respectively. These results demonstrate that while the overall survey is reliable in this pilot study, it can be refined for improvement in future research to ensure even greater reliability. The validity of the questionnaire was also assessed. To achieve this, a thorough review of several established questionnaires in the literature was conducted. Drawing from these sources, the questionnaire was designed in collaboration with domain experts to align with our specific aims and objectives. This process ensured that our instrument accurately captures the necessary data while being tailored to the unique aspects of our study.

### Ethical Considerations

Ethical approval was sought and obtained from the Institutional Review Board at the American University of Beirut (protocol number SBS-2020‐0317) and the respective ethical committees at the Ministry of Public Health and the United Nations Relief and Works Agency. Before participation, HCPs from the recruited centers received an explanation of the study’s objectives, procedures, potential risks (there is no more than the “minimal risk” in participating in this project), and benefits. Consent forms were signed in person, ensuring participants understood the study details and their rights, including their right to withdraw at any point without consequences.

In order to maintain confidentiality and privacy, the gathered data was stored on a password-protected computer only accessible to the research team members. The data was not kept in public archives. All data will be properly destroyed after the required retention period, which is typically 3 years. Participants’ privacy will be secured in all written and published data arising from this study. Names of participants or other identifying information will not be used in reports or published papers.

While no general compensation was provided to study participants for their overall participation, HCPs could receive incentives within the GAIN MHI app’s gamification component based on specific performance metrics. Recognitions, such as the most valuable player or the MIP, were associated with monthly monetary rewards equivalent to US $150.

### Statistical Analysis

Statistical analysis was conducted to derive insights from the quantitative data collected from the cohort of 12 participants who maintained their participation till the end of the study. The initial phase encompassed data management and recoding using Stata MP 17. Subsequently, a descriptive analysis was performed, presenting the frequencies and percentages of various participant characteristics, as well as responses to the survey across all sections. This includes assessment of satisfaction and engagement levels and evaluation of gamification and AI components in the GAIN MHI mobile app. To summarize these sections, mean percentages were calculated, accompanied by SDs and 95% CIs. Concurrently, the qualitative aspect of the study used a coding reliability thematic analysis approach, following a small-q paradigm [45]. This method emphasizes a systematic and transparent methodology to ensure the dependability of the research process, data collection, and analysis. Purposive sampling was used, where qualitative data were collected from all 12 HCPs who used the GAIN MHI app and maintained their participation till the end of the study, regardless of data saturation [46]. Thematic analysis was conducted manually for each of the 5 open-ended questions separately, beginning with an in-depth immersion in the data to gain familiarity with its content. Coding was then performed, wherein responses were systematically labeled with codes, which were subsequently organized into themes. These themes were reviewed iteratively to ensure they accurately represented the data and captured both frequently mentioned ideas and unique perspectives. Finally, a narrative synthesis was developed to integrate all relevant themes. To enhance the confirmability of the study’s findings and interpretations, participants’ direct quotes were incorporated into the analysis. Credibility of the study’s findings was established through prolonged engagement with the data, allowing for a deep understanding of its nuances and depth. Transferability of the study’s findings was addressed through the use of purposeful sampling.

## Results

### Quantitative Analysis

Table 2 provides an overview of participant satisfaction levels with various aspects of the GAIN MHI mobile app. The overall satisfaction percentage calculated is 85% (SD 7%). In terms of the mobile app in general, a majority of participants (10/12, 83%) reported satisfaction, with 17% (n=2) of the participants indicating high satisfaction and no dissatisfaction reported. The user interface of the mobile app showed 50% (n=6) of the participants expressing high satisfaction, 42% (n=5) of the participants expressing satisfaction, and 8% (n=1) of the participants indicating dissatisfaction. All of the surveyed app users were satisfied with the number of MCQs per month, followed by 92% satisfaction with the explanation after each question, the relevance of the links to additional resources, the scoring of the MCQs, and the user interface of the app. The area with comparatively lowest satisfaction is related to the topics covered by the MCQs.

**Table 2.** Satisfaction with gamification, artificial intelligence, and mHealth network for maternal health improvement (GAIN MHI) mobile app.

Variables	Values
Mobile app in general, n (%)	
Highly dissatisfied	0 (0)
Dissatisfied	0 (0)
Neither satisfied nor dissatisfied	0 (0)
Satisfied	10 (83)
Highly satisfied	2 (17)
Number of multiple-choice questions (MCQs) per month (30 questions per month), n (%)	
Highly dissatisfied	0 (0)
Dissatisfied	0 (0)
Neither satisfied nor dissatisfied	0 (0)
Satisfied	9 (75)
Highly satisfied	3 (25)
Topics that the MCQs covered, n (%)	
Highly dissatisfied	0 (0)
Dissatisfied	0 (0)
Neither satisfied nor dissatisfied	2 (17)
Satisfied	4 (33)
Highly satisfied	6 (50)
Explanations after each MCQs encounter, n (%)	
Highly dissatisfied	1 (8)
Dissatisfied	0 (0)
Neither satisfied nor dissatisfied	0 (0)
Satisfied	7 (58)
Highly satisfied	4 (33)
Relevance of the links mentioned in the additional resources, n (%)	
Highly dissatisfied	0 (0)
Dissatisfied	0 (0)
Neither satisfied nor dissatisfied	1 (8)
Satisfied	6 (50)
Highly satisfied	5 (42)
Point/scoring system, n (%)	
Highly dissatisfied	0 (0)
Dissatisfied	0 (0)
Neither satisfied nor dissatisfied	1 (8)
Satisfied	7 (58)
Highly satisfied	4 (33)
User interface of the mobile app, n (%)	
Highly dissatisfied	0 (0)
Dissatisfied	1 (8)
Neither satisfied nor dissatisfied	0 (0)
Satisfied	5 (42)
Highly satisfied	6 (50)
Total satisfaction (%)	
Mean (SD)	85 (7.0)
95% CI	80.5-89.5

Table 3 provides insights into the participants’ levels of engagement with the GAIN MHI mobile app. Participants’ engagement with links from the knowledge resource center was notable, as 50% (n=6) of the participants indicated engaging “Sometimes,” and 33% (n=4) of the participants reported “Often”. Regarding changing clinical routines, 42% (n=5) of the participants reported that they “Often” make adjustments, and 33% (n=4) of the participants did so “Sometimes.” Perceptions of theoretical knowledge improvement due to app usage were generally positive among participants. Specifically, 33% (n=4) of health care providers indicated that their theoretical knowledge "always" increased because of using the GAIN MHI app, 58% (n=7) of health care providers reported that it "often" increased, and 8% (n=1) of health care providers said it "sometimes" did. The total engagement percentage calculated is 65% (SD 6.2%).

**Table 3.** Level of engagement (N=12).

Variables	Values
I accessed the links shared below the MCQs^a^ explanations, n (%)	
Never	0 (0)
Rarely	3 (25)
Sometimes	7 (58)
Often	1 (8)
Always	1 (8)
I visited and accessed links from the knowledge resource center, n (%)	
Never	2 (17)
Rarely	4 (33)
Sometimes	6 (50)
Often	0 (0)
Always	0 (0)
I changed some of my clinical routines and practice because of the overall information captured from the app, n (%)	
Never	1 (8)
Rarely	1 (8)
Sometimes	4 (33)
Often	5 (42)
Always	1 (8)
My theoretical knowledge increased because of using the app, n (%)	
Never	0 (0)
Rarely	0 (0)
Sometimes	1 (8)
Often	7 (58)
Always	4 (33)
Total engagement (%)	
Mean (SD)	64.6 (6.2)
95% CI	60.6-68.5

a MCQ: multiple-choice question.

Table 4 evaluates the gamification and AI components of the GAIN MHI mobile app, revealing strong agreement and satisfaction. Gamification made learning engaging and enjoyable for 92% (n=11) of participants, motivating 92% (n=11) and boosting interest in targeted areas for 100% (n=12). The monetary reward type satisfied all the respondents. Remarkably, the incorporation of gamification components substantially elevated participants’ overall satisfaction with the learning experience (100%). Collectively, participants’ positive responses highlight the success of gamification, with a total average score of 88.5% (SD 7.7%). Moving to the AI components, the findings revealed positive responses (Table 5). The AI adapted to participants’ needs with 83% (n=10) either agreeing or strongly agreeing. Personalization of the learning experience yielded a combined agreement of 75% (n=9). The AI component effectively guided participants to identify their weak areas with an agreement rate of 83%. They expressed full agreement with the enhancement of the overall satisfaction of the learning experience. The cumulative AI score was 82.9% (SD 15.9%). Recommendations were robust, with 100% (n=12) of respondents agreeing on suggesting the app to other colleagues. The total evaluation reached 87.2% (SD 8.9%), solidifying the app’s effectiveness, particularly through its gamification and AI components.

**Table 4.** Evaluation of the gamification, artificial intelligence, and mHealth network for maternal health improvement (GAIN MHI) gamification and artificial intelligence components.

Variables	Values
The gamification component of the GAIN MHI app made learning/professional development enjoyable, fun, and more engaging, n (%)	
Strongly disagree	0 (0)
Disagree	0 (0)
Neither disagree nor agree	1 (8)
Agree	7 (58)
Strongly agree	4 (33)
The gamification component of the GAIN MHI app motivated me to use the app, n (%)	
Strongly disagree	0 (0)
Disagree	0 (0)
Neither disagree nor agree	1 (8)
Agree	4 (33)
Strongly agree	7 (58)
The gamification component of the GAIN MHI app increased my interest in the subject/knowledge areas targeted by the app, n (%)	
Strongly disagree	0 (0)
Disagree	0 (0)
Neither disagree nor agree	0 (0)
Agree	7 (58)
Strongly agree	5 (42)
I was satisfied with the most valuable player (MVP) recognition concept, n (%)	
Strongly disagree	0 (0)
Disagree	0 (0)
Neither disagree nor agree	2 (17)
Agree	5 (42)
Strongly agree	5 (42)
I was satisfied with the most improved player (MIP) recognition concept, n (%)	
Strongly disagree	0 (0)
Disagree	0 (0)
Neither disagree nor agree	2 (17)
Agree	4 (33)
Strongly agree	6 (50)
I felt challenged to showcase my best performance because of the recognition system incorporated in the GAIN MHI mobile app (MVP and MIP), n (%)	
Strongly disagree	1 (8)
Disagree	0 (0)
Neither disagree nor agree	0 (0)
Agree	2 (17)
Strongly agree	9 (75)
I was satisfied with the type of reward offered (cash US $/monetary), n (%)	
Strongly disagree	0 (0)
Disagree	0 (0)
Neither disagree nor agree	0 (0)
Agree	5 (42)
Strongly agree	7 (58)
The gamification component of the GAIN MHI app improved my overall satisfaction with the learning experience, n (%)	
Strongly disagree	0 (0)
Disagree	0 (0)
Neither disagree nor agree	0 (0)
Agree	5 (42)
Strongly agree	7 (58)
Gamification components (%)	
Mean (SD)	88.5 (7.7)
95% CI	83.7-93.4
I have noticed that the GAIN MHI app was focusing on the knowledge areas that I was answering incorrectly the most, n (%)	
Strongly disagree	1 (8)
Disagree	1 (8)
Neither disagree nor agree	0 (0)
Agree	5 (42)
Strongly agree	5 (42)
The AI^a^ component of the GAIN MHI app personalized my learning experience, n (%)	
Strongly disagree	1 (8)
Disagree	1 (8)
Neither disagree nor agree	1 (8)
Agree	4 (33)
Strongly agree	5 (42)
The AI component of the GAIN MHI app helped me better learn about the areas that I needed to learn about, n (%)	
Strongly disagree	0 (0)
Disagree	0 (0)
Neither disagree nor agree	2 (17)
Agree	4 (33)
Strongly agree	6 (50)
The AI component of the GAIN MHI app improved my overall satisfaction with the learning experience, n (%)	
Strongly disagree	0 (0)
Disagree	0 (0)
Neither disagree nor agree	0 (0)
Agree	8 (67)
Strongly agree	4 (33)
Artificial intelligence components (%)	
Mean (SD)	82.9 (15.9)
95% CI	72.83-93
I would recommend the GAIN MHI app to other colleagues, n (%)	
Strongly disagree	0 (0)
Disagree	0 (0)
Neither disagree nor agree	0 (0)
Agree	4 (33)
Strongly agree	8 (67)
Total evaluation (%)	
Mean (SD)	87.2 (8.9)
95% CI	81.6-92.8

a AI: artificial intelligence.

**Table 5.** Mean percentage (%) of health providers’ perception on the role of integrating artificial intelligence in the GAIN MHI mobile app.

Perceived role of artificial intelligence integration	Mean percentage, %
Targeted knowledge areas (yes)	80
Personalized learning experience (yes)	78
Exploring knowledge gaps (yes)	87
Enhanced learning satisfaction (yes)	87

### Qualitative Analysis

The qualitative analysis of users’ favorite aspects of the mobile app reveals several key themes. One respondent mentioned: "I liked the diversity of questions, the focus on wrong answers in the upcoming questions, and the way how the app refreshes our memory.” Another participant noted: “The application was easy to use. The app motivated us to learn more.” Most users appreciate the app’s ease of use and intuitive interface, as well as its ability to facilitate learning and refresh memory on a variety of topics. Furthermore, participants highly value the diversity of content, encompassing a wide range of questions and subject areas. Notably, the app’s motivational elements have been recognized for their capacity to stimulate users in pursuing continuous learning.

Conversely, the analysis has identified potential areas for improvement and enhancement. A total of 4 out of 12 (33%) users reported technical issues that affect the app’s speed and the display of scores. Additionally, suggestions for new features stated, such as the integration of a French language option and enhanced navigation through the Knowledge Resource Center section.

Exploring rewards and incentives, the analysis reveals a general satisfaction with the current monetary rewards. However, suggestions for diversification have emerged, including proposals for increased prizes or trips. A recurring theme is the desire for professional development opportunities, with participants recommending the inclusion of workshops or conferences within the reward system. This sentiment to “organize workshops to improve our work and enhance knowledge sharing” was also expressed.

Delving into content expansion, while some participants expressed contentment with the current topics, others identified gaps related to maternal and reproductive health. Suggestions encompass a range of subjects, spanning from breastfeeding, pregnancy nutrition, family planning, and gynecological diseases, to matters of mental health during pregnancy and emergency case management. Considering the app’s potential extension to other health topics, various priorities have been highlighted. Respondents emphasized chronic conditions, such as diabetes and hypertension. Pediatrics and public health themes like hygiene, food safety, and vaccinations were also suggested. Mental health emerged as a vital area, including discussions on societal issues like gender-based violence and psychological disorders. Moreover, specialized topics like geriatric medicine, emergency medicine, cancer, learning disabilities, autism spectrum disorder, and immunology were proposed. This underscores the breadth of interest and possibilities for future gamified learning programs across diverse health domains.

## Discussion

### Overview

Analysis from this study underscores participants’ overall encouraging experiences with the app, highlighting high levels of satisfaction in various components.

### mLearning Experience With the GAIN MHI App

The findings from this pilot study underscore the keen interest of HCPs in learning and pursuing continuing education. Particularly noteworthy was their engagement with the knowledge resource center. Recent pilot studies have yielded findings that propose a correlation between certain eLearning strategies and the potential equivalence in effectiveness to conventional training methodologies [19,47]. This implies that digital learning approaches, when tailored with specific parallels to traditional training methods, could potentially generate comparable outcomes in terms of skill and knowledge acquisition. The incorporation of the GAIN MHI mLearning app proved instrumental in not only transforming clinical routines but also fostering the enrichment of theoretical knowledge among the HCPs. These findings further validate outcomes from prior research, indicating that using electronic tools is recognized as a potential avenue for enhancing the quality of care provided by HCPs, by increasing their self-efficacy [21,48]. Consequently, such intervention may suggest a possible solution to tackle the issue of insufficient availability of current evidence-based training and reference resources for HCPs working in remote regions.

### Gamification Component of the GAIN MHI App

To our best knowledge, the GAIN MHI app is the first in the Middle East and North Africa region to combine both gamification and AI components in one mobile app to enhance the knowledge and practices of health care workers providing maternal health services. Preliminary evaluations from this study reveal a high level of agreement among HCPs that the gamification component of the GAIN MHI app has made their learning experience more joyful, engaging, and has enhanced their interest in targeted topics. In addition, the users revealed that the gamification component motivated them to use the app with high agreement on the recognition concepts as the most valuable (eg, MIP and monetary prizes). These occurrences might be clarified by the notion that gamification amplifies the spirit of competition through the incorporation of elements like badges, rewards, etc, actively motivating and involving individuals [49-51]. The idea of providing rewards adds to the pleasure of players as they complete levels, enhance skills, and experience progress [52]. This effectively motivates them to sustain their efforts [52]. In line with this strategy, the “Mylearning” platform in the SDA in India was structured to gamify learning and push users to always pursue education and development, seeking the SDA champion title and the provided certification upon successful completion [25]. In the future, gamification will transcend its role as a straightforward application of game-based design elements and evolve into a strategic management tool. It will involve reshaping the cognitive processes, fostering cooperation, and promoting collaboration while innovating novel approaches to learning and community advancement [52].

### Artificial Intelligence Component of the GAIN MHI App

The majority of the HCPs enrolled in this study showed a high desire to recommend the GAIN MHI app to their colleagues and suggested its implication in other medical domains, which highlights the fact that continuing education is essential and applies to different health care practitioners [53]. The potential of AI to customize and personalize the learning experience stands out as one of the most significant potential developments in medical education [53]. A study by Mir et al [53] showed that the implementation of AI-based intervention among medical students empowered them to identify their areas of knowledge gaps and act accordingly. Comparable findings were reported in our study, with 75% of the GAIN MHI app users agreeing on tailoring their learning experience. AI has proved to enhance the HCPs’ learning process and promote continuous advancement within the field [54,55], given that medical education is perceived as a lifetime learning journey [53]. These preliminary evaluations emphasize the importance of integrating AI into interventions targeting the HCPs. Such integration may serve as a valuable tool for their ongoing professional development through continuing education. The results of these early assessments highlight the potential benefits and effectiveness of using AI-driven approaches to enhance the learning experiences and knowledge acquisition of health care professionals by identifying their knowledge gaps. As a result, AI could play a crucial role in shaping how HCPs engage with educational content and adapt their learning strategies based on personalized insights provided by AI technologies. This not only supports their professional growth but also ensures that they stay up-to-date with the latest advancements in their field.

### Lessons Learned and Potential for Scaling Up

The pilot test of the GAIN MHI app yielded insightful lessons with respect to user satisfaction and engagement. A pivotal observation pertained to the app’s innate ability, driven by the synergy of AI and gamification components, to enhance user engagement. The amalgamation of these elements not only captured users’ interest but also fostered a sustained and enthusiastic interaction with the app, primarily attributable to the intrinsic rewards offered through gamified experiences. This underscores the importance of cultivating a personalized and engaging environment to ensure continued user participation and commitment, in order to contribute to the professional development of the HCPs. Furthermore, the pilot test revealed that users were not only inclined to use the GAIN MHI app for their own professional development but also recommended it to their colleagues within the health care community, which underscores its potential as a valuable tool for broader adoption. Users appreciated the convenience of the mobile platform, the data-driven insights offered by the AI component, and the motivation instilled by the gamified structure. Moreover, their expressed interest in exploring additional topics beyond the current scope of the app indicates a desire for diverse and comprehensive educational content. This highlights the app’s potential to serve as a versatile and adaptable platform catering to a wide spectrum of health care professionals’ learning needs. This preliminary assessment also garnered important feedback regarding the improvement of the content of the app, both in terms of the asked MCQs and the associated Knowledge Resource Center. This feedback is guiding improvements in the design and content of the app, which would be vital for future upscaling. Future work will focus on refining the content and design of the GAIN MHI app based on user feedback, particularly in enhancing the quality of MCQs. This process is crucial for ensuring the continued engagement and satisfaction of users, ultimately contributing to the professional development of HCPs and facilitating broader adoption of the app within the health care community in Lebanon.

### Limitations

While this pilot study offers valuable insights into the satisfaction of HCPs using the GAIN MHI app in improving maternal health outcomes among pregnant women residing in a fragile setting, it is important to acknowledge several limitations that were encountered during the course of this investigation. One primary constraint revolved around the limited sample size, consisting of merely 12 [12] HCPs located within the selected centers. This constraint may shed light on the possibility of a shortage of HCPs in these centers, potentially influencing the representation of provider perspectives and experiences in the study. Another limitation faced during the study was the consecutive malfunction and slowness of the app due to network issues. To address this concern, the implementation of an offline version of the app could potentially offer a viable solution for improving display speed and ensuring seamless accessibility to the app, especially in remote areas. Last, this GAIN MHI app version was developed in 2 languages: Arabic and English. However, it did not take into consideration French-educated HCPs. This concern could be rectified by upgrading the GAIN MHI app to include other languages in the future.

### Conclusions

HCPs, especially those working in remote and underserved areas, find it increasingly difficult to engage in professional development activities. Embarking on modern techniques and pedagogies is essential to empower them to learn at a time and place of their convenience. Personalizing the learning experience and enhancing participation through gamification are the future of education.

The consensus among HCPs emphasizes a high level of satisfaction with the GAIN MHI app and its integrated gamification and AI components. The positive reception reaffirms the potential of incorporating such innovative approaches to facilitate the advancement of their professional skills. The findings of this study build a strong case for upscaling similar apps into various contexts and specialties. In doing that, it is recommended that future studies delve deeper into assessing the app’s feasibility and reception across a broader spectrum of HCPs and contexts, alongside its effect on maternal health outcomes.

## Abbreviations:

AI: artificial intelligence
GAIN MHI: gamification, artificial intelligence, and mHealth network for maternal health improvement
HCP: health care provider
MCQ: multiple-choice question
mHealth: mobile health
MIP: most improved player
OBGYN: obstetrics and gynecology
PHC: primary health care center
SDA: Safe Delivery App

## References

[1] (2023). Maternal mortality. WHO.

[2] El‐Kak F, Kabakian‐Khasholian T, Ammar W, Nassar A (2020). A review of maternal mortality trends in Lebanon, 2010–2018. Int J Gynaecol Obstet.

[3] Geller SE, Koch AR, Garland CE, MacDonald EJ, Storey F, Lawton B (2018). A global view of severe maternal morbidity: moving beyond maternal mortality. Reprod Health.

[4] Reese Masterson A, Usta J, Gupta J, Ettinger AS (2014). Assessment of reproductive health and violence against women among displaced Syrians in Lebanon. BMC Womens Health.

[5] Talhouk R, Mesmar S, Thieme A (2016). Syrian refugees and digital health in Lebanon: opportunities for improving antenatal health.

[6] Carmichael SL, Mehta K, Srikantiah S (2019). Use of mobile technology by frontline health workers to promote reproductive, maternal, newborn and child health and nutrition: a cluster randomized controlled trial in Bihar, India. J Glob Health.

[7] Jaeger FN, Bechir M, Harouna M, Moto DD, Utzinger J (2018). Challenges and opportunities for healthcare workers in a rural district of Chad. BMC Health Serv Res.

[8] Mormina M, Pinder S (2018). A conceptual framework for training of trainers (ToT) interventions in global health. Global Health.

[9] El-Jardali F, Alameddine M, Jamal D (2013). A national study on nurses’ retention in healthcare facilities in underserved areas in Lebanon. Hum Resour Health.

[10] Jaana M, Majdalani M, Tamim H, Rahbany R (2018). Perceived healthcare workforce needs in Lebanon: a step towards informed human resources planning and professional development. East Mediterr Health J.

[11] Bou Sanayeh E, El Chamieh C (2023). The fragile healthcare system in Lebanon: sounding the alarm about its possible collapse. Health Econ Rev.

[12] Hachoumi N, Eddabbah M, El Adib AR (2023). Health sciences lifelong learning and professional development in the era of artificial intelligence. Int J Med Inform.

[13] Maheu-Cadotte MA, Cossette S, Dubé V (2018). Effectiveness of serious games and impact of design elements on engagement and educational outcomes in healthcare professionals and students: a systematic review and meta-analysis protocol. BMJ Open.

[14] Gentry SV, Gauthier A, L’Estrade Ehrstrom B (2019). Serious gaming and gamification education in health professions: systematic review. J Med Internet Res.

[15] Feng JY, Chang YT, Chang HY, Erdley WS, Lin CH, Chang YJ (2013). Systematic review of effectiveness of situated e‐learning on medical and nursing education. Worldviews Evid Based Nurs.

[16] Panda M, Desbiens NA (2010). An “education for life” requirement to promote lifelong learning in an internal medicine residency program. J Grad Med Educ.

[17] Rugh JD, Hendricson WD, Hatch JP, Glass BJ (2010). The San Antonio CATs initiative. J Am Coll Dent.

[18] Weisman A, Lin E, Yona T, Gottlieb U, Impellizzeri FM, Masharawi Y (2023). Healthcare providers have insufficient up-to-date knowledge of lower limb sports injuries, and their knowledge is similar to that of athletes. Musculoskelet Sci Pract.

[19] Nilsson C, Sørensen BL, Sørensen JL (2014). Comparing hands-on and video training for postpartum hemorrhage management. Acta Obstet Gynecol Scand.

[20] Sondaal SFV, Browne JL, Amoakoh-Coleman M (2016). Assessing the effect of mHealth interventions in improving maternal and neonatal care in low- and middle-income countries: a systematic review. PLoS One.

[21] Bolan NE, Sthreshley L, Ngoy B (2018). mLearning in the Democratic Republic of the Congo: a mixed-methods feasibility and pilot cluster randomized trial using the Safe Delivery App. Glob Health Sci Pract.

[22] Lund S, Boas IM, Bedesa T, Fekede W, Nielsen HS, Sørensen BL (2016). Association between the Safe Delivery App and quality of care and perinatal survival in Ethiopia: a randomized clinical trial. JAMA Pediatr.

[23] Thomsen CF, Barrie AMF, Boas IM (2019). Health workers’ experiences with the Safe Delivery App in West Wollega Zone, Ethiopia: a qualitative study. Reprod Health.

[24] (2022). Maternity foundation and UNFPA launch new Arabic language version of a popular digital health tool to improve quality training of midwives in the Arab region. UNFPA.

[25] Singh Sodha T, Grønbæk A, Bhandari A, Mary B, Sudke A, Smith LT (2022). mHealth learning tool for skilled birth attendants: scaling the Safe Delivery App in India. BMJ Open Qual.

[26] Al-Rayes S, Al Yaqoub FA, Alfayez A (2022). Gaming elements, applications, and challenges of gamification in healthcare. Inform Med Unlock.

[27] Akl EA, Sackett KM, Erdley WS (2013). Educational games for health professionals. Cochrane Database Syst Rev.

[28] Damaševičius R, Maskeliūnas R, Blažauskas T (2023). Serious games and gamification in healthcare: a meta-review. Information.

[29] Mesko B (2017). The role of artificial intelligence in precision medicine. Expert Rev Precis Med Drug Dev.

[30] Johnson KB, Wei WQ, Weeraratne D (2021). Precision medicine, AI, and the future of personalized health care. Clin Transl Sci.

[31] Brault N, Saxena M (2021). For a critical appraisal of artificial intelligence in healthcare: the problem of bias in mHealth. J Eval Clin Pract.

[32] Zhang S, Bamakan SMH, Qu Q, Li S (2019). Learning for personalized medicine: a comprehensive review from a deep learning perspective. IEEE Rev Biomed Eng.

[33] Haenlein M, Kaplan A (2019). A brief history of artificial intelligence: on the past, present, and future of artificial intelligence. Calif Manage Rev.

[34] Mishra S, Tripathi AR (2021). AI business model: an integrative business approach. J Innov Entrep.

[35] Mustak M, Salminen J, Plé L, Wirtz J (2021). Artificial intelligence in marketing: topic modeling, scientometric analysis, and research agenda. J Bus Res.

[36] Shaheen MY (2021). Applications of artificial intelligence (AI) in healthcare: a review. ScienceOpen Preprints.

[37] Amann J, Blasimme A, Vayena E, Frey D, Madai VI, Precise4Q consortium (2020). Explainability for artificial intelligence in healthcare: a multidisciplinary perspective. BMC Med Inform Decis Mak.

[38] Hosny A, Aerts HJWL (2019). Artificial intelligence for global health. Science.

[39] Rittenhouse KJ, Vwalika B, Keil A (2019). Improving preterm newborn identification in low-resource settings with machine learning. PLoS One.

[40] Araya J, Rodriguez A, Lagos-SanMartin K (2021). Maternal thyroid profile in first and second trimester of pregnancy is correlated with gestational diabetes mellitus through machine learning. Placenta.

[41] Debata PP, Mohapatra P (2020). Diagnosis of diabetes in pregnant woman using a Chaotic-Jaya hybridized extreme learning machine model. J Integr Bioinform.

[42] Jiménez-Serrano S, Tortajada S, García-Gómez JM (2015). A mobile health application to predict postpartum depression based on machine learning. Telemed J E Health.

[43] Natarajan S, Prabhakar A, Ramanan N, Bagilone A, Siek K, Connelly K Boosting for postpartum depression prediction.

[44] Chen M, Decary M (2020). Artificial intelligence in healthcare: an essential guide for health leaders. Healthc Manage Forum.

[45] Braun V, Clarke V (2023). Toward good practice in thematic analysis: avoiding common problems and be(com)ing a knowing researcher. Int J Transgend Health.

[46] Hennink M, Kaiser BN (2022). Sample sizes for saturation in qualitative research: a systematic review of empirical tests. Soc Sci Med.

[47] Dunleavy G, Nikolaou CK, Nifakos S, Atun R, Law GCY, Tudor Car L (2019). Mobile digital education for health professions: systematic review and meta-analysis by the digital health education collaboration. J Med Internet Res.

[48] Walker DM, Holme F, Zelek ST (2015). A process evaluation of PRONTO simulation training for obstetric and neonatal emergency response teams in Guatemala. BMC Med Educ.

[49] King D, Greaves F, Exeter C, Darzi A (2013). “Gamification”: influencing health behaviours with games. J R Soc Med.

[50] Wiley K, Vedress S, Mandryk RL (2020). How points and theme affect performance and experience in a gamified cognitive task.

[51] Fiş Erümit S, Karakuş Yılmaz T (2022). Gamification design in education: what might give a sense of play and learning?. Technol Know Learn.

[52] Ansari S, Khot R, Shaikh Z, Yadav P, Pirani Z (2022). A comprehensive review of gamification in healthcare: incentives in mobile healthcare app. Int Res J Eng Technol.

[53] Mir MM, Mir GM, Raina NT (2023). Application of artificial intelligence in medical education: current scenario and future perspectives. J Adv Med Educ Prof.

[54] Dave M, Patel N (2023). Artificial intelligence in healthcare and education. Br Dent J.

[55] Jacobs SM, Lundy NN, Issenberg SB, Chandran L (2023). Reimagining core entrustable professional activities for undergraduate medical education in the era of artificial intelligence. JMIR Med Educ.

